# Supplementary material for: Morphology, Phenotype, and Molecular Identification of Clinical and Environmental Fusarium solani Species Complex Isolates from Malaysia
Source: J Fungi (Basel). 2022 Aug 11;8(8):845. doi: 10.3390/jof8080845 (PMC9409803; doi:10.3390/jof8080845)
Supplement: Supplementary file 1 [file jof-08-00845-s001.zip › jof-1793805-supplementary.pdf]

Table S1. Primers used for PCR amplification of *Fusarium TEF1-α* and *RPB2* genes.

Locus	Primer		
	Amplicon size (bp)[a]	Name[b]	Nucleotide sequence (5' to 3')[c]
TEF1-α	~700	EF1-F	ATGGGTAAGGARGACAAGAC
		EF2-R	GGARGTACCAGTSATCATG
		EF3-F*	GTAAGGAGGASAAGACTCACC
		EF22T-R*	AGGAACCCTTACCGAGCTC
RPB2	1750	5f2-F	GGGGWGAYCAGAAGAAGGC
		7cr-R	CCCATRGCTTGYTTRCCCAT
		7cf-F	ATGGGYAARCAAGCYATGGG
		11ar-R	GCRTGGATCTTRTCRTCSACC

[a] Final fragment length obtained; *RPB2* consists of two concatenated DNA fragments.

[b] Forward and reverse primers are indicated by -F and -R, respectively; asterisks (*) indicate internal primers that were used only for DNA sequencing of the PCR product amplified using the EF1-F and EF2-R primer pair.

[c] R, A or G; S, C or G; W, A or T; Y, C or T.

Table S2. Source, species and MLST of 37 *Fusarium* isolates selected for the phylogenetic analysis of *TEF1-α* and *RPB2* sequences presented in Figure 1.

**Strain No.**[a]	**Source**	**Country**	**Species**	**MLST type**[b]	**GenBank accession number**	
					TEF1-α	***RPB2***
NRRL 28546	Human cornea	USA	*F. petroliphilum*	1-a	DQ246887	EU329544
NRRL 54986	Unknown	USA	*F. petroliphilum*	1-a	KC808208	KC808348
NRRL 43458	Human	Singapore	*F. keratoplasticum*	2-f	DQ790511	DQ790599
CBS 490.63	Human	Japan	*F. keratoplasticum*	2-d	LT906670	LT960562
NRRL 32719	Human eye	USA	*F. falciforme*	3+4-oo	DQ247039	EU329600
NRRL 22660	*Trichosanthes dioica*	India	*F. falciforme*	3+4-ii	JX435158	JX435258
NRRL 54219	Human spine	USA	*F. falciforme*	3+4-tt	HQ401721	HQ401723
NRRL 66304	*Solanum tuberosum*	Slovenia	*F. solani* sensu stricto	5-j	KT313611	KT313623
NRRL 32484	Human	USA	*F. solani* sensu stricto	5-j	DQ246982	EU329583
NRRL 43489	Human cornea	USA	*F. metavorans*	6-b	DQ790484	DQ790572
NRRL 37640	Human	Turkey	*F. metavorans*	6-f	FJ240355	EU329638
NRRL 32770	Human eye	USA	*F. waltergamsii*	7-d	DQ247083	EU329615
NRRL 32794	Coolant fluid	USA	*F. waltergamsii*	7-c	DQ247103	EU329622
NRRL 43467	Human	USA	*F. neocosmosporiellum*	8-a	EF452940	EF469979
NRRL 34174	Human	USA	*F. neocosmosporiellum*	8-d	AY381145	EU329636
NRRL 32755	Turtle head lesion	USA	*F. tonkinense*	9-a	DQ247073	EU329613
NRRL 43811	Human cornea	USA	*F. tonkinense*	9-a	EF453053	EF470092
NRRL 22098	Cucurbit	USA	*Fusarium* sp.	10-b	AF178327	EU329489
NRRL 22153	Cucurbit	Panama	*Fusarium* sp.	10-a	AF178346	EU329492
NRRL 22278	Pisum sativum	USA	*Fusarium solani f. sp. pisi*	11-c	AF178337	EU329501
NRRL 22820	Glycine max	USA	*Fusarium solani f. sp. pisi*	11-c	AF178355	EU329532
NRRL 22642	*Penaceous japonicus*	Japan	*Fusarium* sp.	12-a	DQ246844	EU329522
NRRL 22834	Lobster	Australia	*Fusarium* sp.	12-a	DQ247663	FJ240382
NRRL 32434	Human	Germany	*F. lichenicola*	16-b	DQ246977	EF470146
NRRL 34123	Human eye	India	*F. lichenicola*	16-b	DQ247192	EU329635
NRRL 32858	Human wound	USA	*F. suttonianum*	20-d	DQ247163	EU329630
CBS 124892	Human nail	Gabon	*F. suttonianum*	20-a	JX435139	JX435239
NRRL 22101	Cotton cloth	Panama	*F. striatum*	21-a	AF178333	EU329490
NRRL 52699	*Mahanarva andigena*	Colombia	*F. striatum*	21-c	JF740782	JF741108
NRRL 37625	Human foot	The Netherlands	*F. cyanescens*	27-a	FJ240353	EU329637
NRRL 37626	Human	The Netherlands	*F. cyanescens*	27-a	FJ240354	FJ240406
NRRL 31757	Glycine max	Brazil	*F. brasiliense*	Clade 2	EF408409	EU329565
CBS 119594	Dead branch	Sri Lanka	*F. mahasenii*	Clade 2	DQ247513	LT960563
NRRL 22632	*Hoheria glabrata*	New Zealand	*F. plagianthi*	Clade 1	AF178354	JX171614
NRRL 22090	*Beilschmiedia tawa*	New Zealand	*F. illudens*	Clade 1	AF178326	JX171601
CBS 125552	*Buxus sempervirens*	Slovenia	*F. cicatricum*	Outgroup	HM626644	HQ728153
NRRL 22316	*Staphylea trifolia*	USA	*F. staphyleae*	Outgroup	AF178361	JX171609

[a] Strains from the United States of America Agricultural Research Service culture collection (NRRL) and the Westerdijk Fungal Biodiversity Institute collection (CBS).

[b] The MLST was determined based on polyphasic identification using the *Fusarium* MLST database.
